# Pro-BDNF Contributes to Hypoxia/Reoxygenation Injury in Myocardial Microvascular Endothelial Cells: Roles of Receptors p75^NTR^ and Sortilin and Activation of JNK and Caspase 3

**DOI:** 10.1155/2018/3091424

**Published:** 2018-06-26

**Authors:** Fei Yu, Yuezhu Liu, Junmei Xu

**Affiliations:** Department of Anesthesiology, The Second Xiangya Hospital of Central South University, Changsha, Hunan 410011, China

## Abstract

The aim of this study was to identify the role of the precursor of the brain-derived neurotrophic factor (pro-BDNF) in myocardial hypoxia/reoxygenation injury (H/R) and to address the underlying mechanisms. For this purpose, myocardial microvascular endothelial cells (MMECs) exposed to a high concentration of glucose (30 mM) for 48 h were subjected to 4 h of hypoxia followed by 2 h of reoxygenation. Terminal deoxynucleotidyl transferase (TdT) dUTP nick-end labeling (TUNEL) staining and flow-cytometric analysis were performed to detect apoptosis. Cell scratch and capillary-like-structure formation assays were employed to evaluate cell function. The levels of apoptosis-related proteins were evaluated by Western blotting and immunofluorescence assays. Our results showed that H/R resulted in MMEC injury, as indicated by significant increases in TUNEL-positive cell numbers and a reduction in MMEC migration and in capillary-like-structure formation coupled with increased pro-BDNF protein expression. In addition, overexpression of pro-BDNF in MMECs via a viral vector led to increased pro-BDNF expression, and this upregulation induced apoptosis. Mechanistic experiments revealed that H/R did not influence BDNF, JNK, and caspase 3 expression, but upregulated pro-BDNF, p75^NTR^, sortilin, phospho-JNK, and cleaved caspase 3 protein levels. In contrast, neutralization of endogenous pro-BDNF with an antibody significantly attenuated H/R-induced upregulation of pro-BDNF, p75^NTR^, sortilin, p-JNK, and cleaved caspase 3 protein levels, indicating that p75^NTR^-sortilin signaling and activation of JNK and caspase 3 may be involved in these effects. In conclusion, H/R-induced injury may be mediated by pro-BDNF, at least in part through the regulation of p75^NTR^-sortilin signaling and activation of JNK and caspase 3.

## 1. Introduction

Diabetes mellitus (DM), a potent and prevalent risk factor of ischemic heart disease, has received increasing attention globally. Cardiovascular complications constitute the leading cause of morbidity and mortality among patients with DM [1–4]. In addition, DM increased myocardial susceptibility to ischemia/reperfusion- (I/R-) caused irreversible destruction, characterized by deficient oxygen supply and subsequent restoration of blood flow [5–8]. Microvascular disturbances are a vital feature of myocardial reperfusion injury [9]. Myocardial I/R is associated with cardiomyocyte apoptosis, infiltration by immune cells, an inflammatory cytokine release, and angiogenesis [10–12]. Cardiac microvascular endothelial cells, a basic component of myocardial microcirculation, were first harmed by reperfusion injury followed by damage to cardiomyocytes after restoration of the cardiac microcirculation and played a vital role in the preservation of cardiomyocytes after reperfusion injury [9, 13]. Moreover, numerous studies have shown that endothelial cell (EC) dysfunction, an important event in virtually all forms of I/R injury, determines the degree of cellular injury after I/R [14]. Nevertheless, the potential mechanisms responsible for the adverse effects caused by apoptosis and endothelial dysfunction after endothelial injury induced by hyperglycemia with I/R insults remain an enigma.

In recent years, studies on the nerve growth factor (NGF) family have been focused on the nervous system [15]. Lately, a large number of studies confirmed that this family also has an important role in the cardiovascular system [16]. The brain-derived neurotrophic factor (BDNF), a member of the NGF family, has been shown to have an antiapoptotic effect against the toxicity of tumor necrosis factor *α* (TNF-*α*) in human microvascular ECs [17]. Pro-BDNF, a precursor of BDNF, has been originally described as a proapoptotic ligand in the nervous system. Thus, it is believed that pro-BDNF may exert proapoptotic action on ECs [18, 19].

Both ECs and endothelial progenitor cells express high-affinity receptors called Trk [20]. NGF and BDNF promoted the growth and angiogenesis of ECs through their high-affinity receptors (TrkA and TrkB) [21]. Besides, NGF promoted the survival and functional recovery of cardiomyocytes after myocardial I/R injury via paracrine pathways [22]. P75^NTR^, a low-affinity receptor for neurotrophins, is involved in a diverse array of cellular responses, including apoptosis. Sortilin is known as a coreceptor of p75^NTR^, and its deficiency is reported to reduce apoptosis [23]. The actions of pro-BDNF are mediated by a receptor complex of p75^NTR^ and sortilin [24]. Pro-BDNF with high affinity for p75^NTR^ may be deeply involved in myocardial ischemia/reperfusion injury (MIRI). c-Jun N-terminal kinase (JNK) is indispensable for both cell proliferation and apoptosis. However, the molecular mechanism that underlies the participation of pro-BDNF in the process of endothelial I/R-induced apoptosis has not been elucidated completely.

JNK, one of the members of the MAP kinase superfamily, is primarily involved in the induction of death receptor-initiated exogenous and mitochondrial apoptosis in vivo after exposure to various chemical or biological agents [25]. JNK activated apoptotic signaling pathways by transactivation of specific transcription factors or by modulating the activity of mitochondrial proapoptotic and antiapoptotic proteins directly through different phosphorylation events, thereby increasing proapoptotic gene expression [26]. Activated JNK, in turn, phosphorylated c-Jun and proteins associated with apoptosis such as caspase 3 [27]. Caspase 3 activity is a biochemical hallmark of apoptosis, and imaging the activity is a part of an assay in an apoptosis-targeted treatment response in cancer [28]. Regulation of the activity of the JNK signaling pathway is vital for protecting myocardial cells from I/R injury [29, 30].

Currently, there is no evidence that pro-BDNF participates in the process of endothelial I/R injury, and the corresponding molecular mechanism is unclear. In the present study, we investigated the role of pro-BDNF in the regulation of hypoxia/reoxygenation- (H/R-) induced endothelial apoptosis, migration, and tube formation and next examined the expression of proteins related to apoptosis. These findings will lead to a novel therapeutic approach for myocardial I/R injury.

## 2. Materials and Methods

### 2.1. Cell Culture

The human myocardial microvascular endothelial cell (MMEC) line was purchased from the American Type Culture Collection (ATCC, Rockville, MD, USA) and maintained in the DMEM high-glucose complete medium (Gibco, Waltham, MA, USA), supplemented with 10% of fetal bovine serum (HyClone, Logan, UT, USA), 100 U/mL penicillin (Sigma, St. Louis, MO, USA), and 100 *μ*g/mL streptomycin (Sigma) in a humidified atmosphere containing 5% of CO_2_ at 37°C. MMECs (passages 3 to 5), characterized by typical cobblestone appearance and by positive CD31 and CD34 immunostaining [31], were used for the following experimental analysis.

### 2.2. H/R Injury Induction

To induce H/R injury as described previously [32], an I/R model was established by means of MMECs. The cells were incubated in a high-glucose culture medium (30 mM) for 48 h and then exposed to hypoxia (5% CO_2_, 1% O_2_, and 94% N_2_) for 4 h followed by 2 h of reoxygenation (5% CO_2_, 21% O_2_, and 94% N_2_).

### 2.3. Viral-Vector Transduction of MMECs and Antibody Neutralization

The recombinant adenoviruses expressing the human pro-BDNF gene (Ad-GFP-pro-BDNF) or GFP control (Ad-GFP) were purchased from GenePharma (Shanghai, China) and were used to infect the ECs according to the manufacturer's instructions. Transduction efficiency was verified via GFP expression and Western blotting. The neutralizing antibody to the recombinant prodomain of BDNF (10 *μ*g/mL), specifically recognizing pro-BDNF but not mature BDNF or other neurotrophins, was added into the culture medium prior to induction of H/R [33–36].

### 2.4. Apoptosis Assay

Apoptosis was detected by the TUNEL assay (Roche Applied Science) and by corresponding flow-cytometric analyses according to the instructions of the manufacturer. For quantification, the TUNEL-positive cells were counted in at least five randomly chosen visual fields in three independent samples (500 counted cells in total). The flow-cytometric assay was then performed on a BD FACSCalibur instrument (Becton, Dickinson and Company, Lake Franklin, NJ, USA).

### 2.5. Western Blot Analysis

This analysis was conducted to determine the protein expression and phosphorylation levels. Cellular proteins were extracted with RIPA lysis buffer (Beyotime Institute of Biotechnology). Proteins (lysate corresponding to 20 *μ*g of protein) were loaded onto a gel and separated in each lane by sodium dodecyl sulfate polyacrylamide gel electrophoresis (SDS-PAGE) lasting for 2 h at 100 V in a buffer and were transferred to polyvinylidene fluoride (PVDF) membranes. After blocking in 5% fat-free dry milk, antibodies against pro-BDNF (Alomone, 1 : 400), BDNF (Abcam, 1 : 500), P75 (Santa Cruz Biotechnology, Santa Cruz, CA, USA; 1 : 100), sortilin (Abcam, 1 : 500), JNK (Santa Cruz Biotechnology, 1 : 100), cleaved caspase 3 (Asp175, Cell Signaling Technology, 1 : 1000), caspase 3, and *β*-actin (Santa Cruz Biotechnology) were employed. Antibody binding was detected by chemiluminescence with a Tanon 5500 Imaging System (Tanon Science & Technology Ltd., Shanghai, China) and quantified in the ImageJ software (NIH, Bethesda, MD, USA).

### 2.6. Immunofluorescence Analysis

Cells were fixed with 4% paraformaldehyde at room temperature (RT) for 30 min and permeabilized or not permeabilized with 0.5% Triton X-100 (Sigma-Aldrich), and nonspecific binding was blocked by incubation with 5% donkey serum (Jackson ImmunoResearch Laboratories) at RT for 30 min. Coverslips were incubated overnight at 4°C with the following primary antibodies: rabbit anti-BDNF (Abcam, 1 : 500), rabbit anti-pro-BDNF (Alomone Labs, 1 : 400), anti-JNK (Santa Cruz Biotechnology, 1 : 100), anti-phosphorylated-JNK (p-JNK) (Santa Cruz Biotechnology, 1 : 100), rabbit anti-p75^NTR^ (Santa Cruz Biotechnology, 1 : 100), and goat anti-sortilin (Abcam, 1 : 500) antibodies. A secondary antibody conjugated with Cy3 or fluorescein isothiocyanate was incubated for 2 h at RT. Nuclei were stained for 5 min with 4′,6-diamidino-2-phenylindole (DAPI). Cells were washed three times in PBST after each incubation. Pictures were taken using a confocal microscope (Carl Zeiss, LSM 510).

### 2.7. Assays of Capillary-Like-Structure Formation and Cell Scratches In Vitro

We performed a cell scratch assay and capillary-like-structure formation experiments to evaluate the functional effects of pro-BDNF on MMECs in groups control, H/R, H/R + anti-pro-BDNF, and H/R + vehicle.

The assay of capillary-like-structure formation in vitro was performed as previously described [37]. Briefly, ECs (10^5^/well) were cultured in a 24-well plate coated with 200 *μ*L of Matrigel (356234; BD Biosciences). Capillary-like-structure formation was imaged after 12 h in five random microscopic visual fields by means of an inverted phase contrast microscope. The cell scratch assay was conducted to detect the migration of MMECs [38]. For the scratch assay, MMECs were cultured until confluence. After serum starvation for 24 h, a linear wound was administered by scratching the bottom of the dish with a pipette tip. The wound images were captured 24 h after scratching using a Motic AE31 Photometry and Dimensioning microscope (Milton, MA, USA).

### 2.8. Statistical Analysis

All values are presented as means ± standard error of the mean (SEM). Statistical analysis was performed by one-way ANOVA to compare multiple groups and by Student's *t*-test to compare two groups. Data with *P* < 0.05 were considered statistically significant. Statistical analysis was performed in the SPSS Statistics software (version 16.0).

## 3. Results

### 3.1. H/R Induces Apoptosis with Upregulation of Pro-BDNF in MMECs Exposed to High Concentration of Glucose

**Figure 1:**
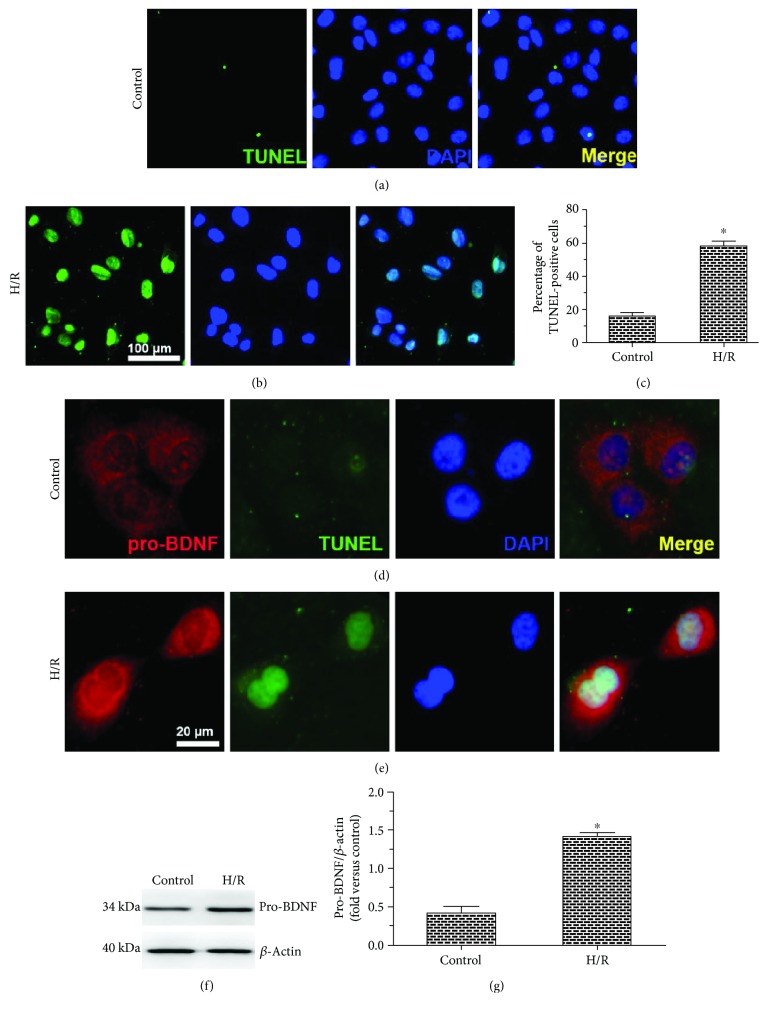
Effects of H/R on the apoptosis and pro-BDNF expression among MMECs exposed to HG. (a, b) Representative images of the TUNEL assay of MMECs exposed to HG without (control) or with (H/R group) H/R. (c) The percentage of TUNEL-positive cells. H/R significantly increased the percentage of TUNEL-positive cells among MMECs, indicating the induction of apoptosis. (d, e) Immunostaining results on the pro-BDNF protein expression and a TUNEL assay. (f, g) Representative Western blots and quantitative analysis of pro-BDNF protein. H/R markedly increased the expression of pro-BDNF. The data were analyzed by the *t*-test. The error bars represent SEM. ^∗^*P* < 0.05 as compared with the control group.

We first examined the effects of H/R on MMECs after exposure to high concentration of glucose (HG). Representative photographs were taken, and quantitative analysis of TUNEL positivity was performed to evaluate the proapoptotic effects. After exposure to HG, H/R caused a significant increase in the proportion of TUNEL-positive cells as compared to MMECs not subjected to H/R (control group), indicating that H/R induced MMEC apoptosis (Figures 1(a)–1(c)).

Next, we examined the effect of H/R on pro-BDNF protein levels. The expression of pro-BDNF measured by immunostaining was observed in the cytoplasm and plasma membrane of MMECs. Of note, exposure to H/R caused overlapping signals of pro-BDNF staining and TUNEL staining among MMECs (Figures 1(d) and 1(e)), together with higher levels of pro-BDNF as measured by Western blot analysis in comparison with controls (Figures 1(f) and 1(g)). These results indicate that H/R exerted a proapoptotic effect and upregulated the pro-BDNF protein.

### 3.2. Pro-BDNF Overexpression Promotes MMEC Apoptosis

**Figure 2:**
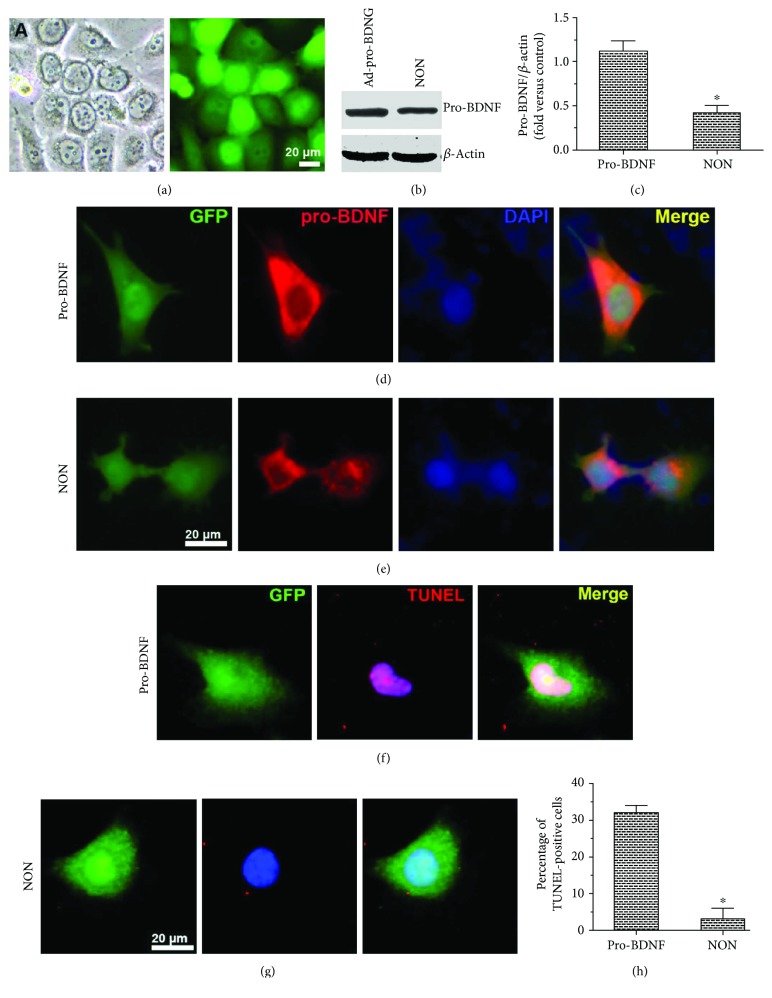
Overexpression of pro-BDNF in MMECs and its effect on MMEC apoptosis. (a–e) MMECs were transfected with either pro-BDNF or Ad-GFP. Immunostaining, Western blotting, and quantitative analysis showed that the protein expression of pro-BDNF increased in MMECs after transduction with pro-BDNF. (f–h) Transfected cells were exposed to HG and then subjected to a TUNEL assay (f, g) and enumeration of TUNEL-positive cells (h) to evaluate apoptosis. Pro-BDNF overexpression markedly elevated the numbers of TUNEL-positive cells. The data were analyzed by the *t*-test. The error bars represent SEM. ^∗^*P* < 0.05 as compared with the control group or Ad-pro-BDNF group.

To test whether an increase in pro-BDNF levels exerted proapoptotic actions on MMECs under HG conditions, we transfected MMECs with either Ad-pro-BDNF or with Ad-GFP as a negative control group (NON). A TUNEL assay of adenovirus-infected MMECs under HG conditions was then performed (Figures 2(a)–2(e)). The protein expression of pro-BDNF significantly increased after transduction with Ad-pro-BDNF as determined by immunostaining and Western blot analysis, as compared with that in Ad-GFP-transfected cells (NON). In addition, Ad-pro-BDNF-transfected MMECs showed a significant increase in the number of TUNEL-positive cells (Figures 2(f)–2(h)). In short, MMEC apoptosis was induced by pro-BDNF.

### 3.3. Pro-BDNF Is Required for H/R-Induced Apoptosis and Dysfunction

**Figure 3:**
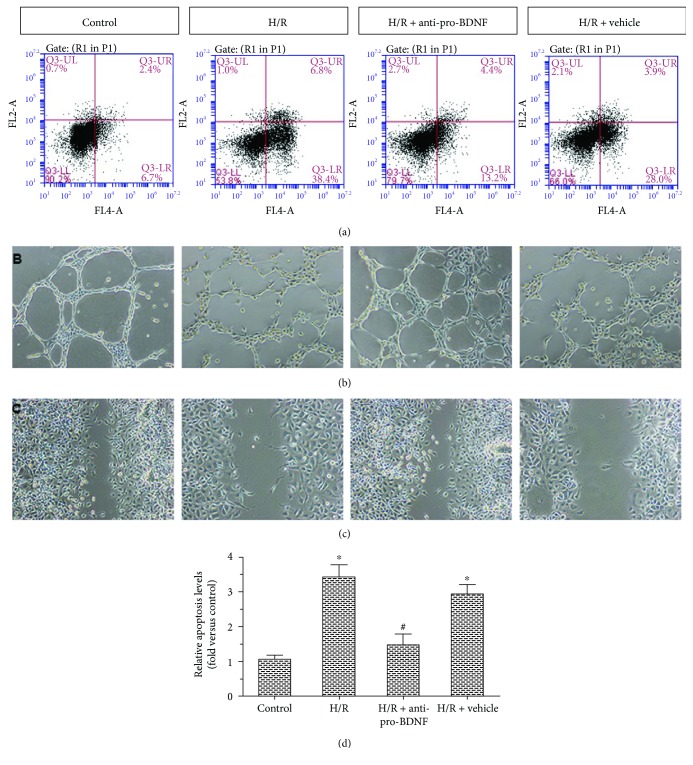
Effects of the anti-pro-BDNF antibody on apoptosis, migration, and capillary-like-structure formation among MMECs after exposure to HG and H/R. (a) Effects of pro-BDNF on apoptosis were analyzed by flow cytometry of MMECs after different treatments: control, H/R, H/R + anti-pro-BDNF, and H/R + vehicle. (b, c) The functional effects of pro-BDNF on MMECs were assessed by capillary-like-structure formation and cell scratch assays. (d) Relative apoptosis levels and fold changes are expressed in relation to the control group. The H/R group showed markedly increased relative apoptosis levels, decreased capillary-like-structure formation, and reduced cell migration when compared with the control group. These effects were reversed by treatment with the anti-pro-BDNF antibody to the levels similar to those in the control group. The data were subjected to one-way ANOVA. The error bars represent SEM. ^∗^*P* < 0.05 as compared with the control group; ^#^*P* < 0.05 as compared with the H/R group or the H/R + vehicle group.

The proapoptotic action of H/R seemed to be mediated at least in part by upregulation of pro-BDNF. We next evaluated the relation between pro-BDNF and H/R-induced apoptosis (Figures 3(a) and 3(d)). Exposure of MMECs to H/R caused a significant increase in relative apoptosis levels. These effects were abrogated by the exogenous anti-pro-BDNF antibody. These results indicate that H/R could induce MMEC apoptosis by upregulating pro-BDNF.

To address the functional effects of pro-BDNF on MMECs, capillary-like-structure formation experiments (Figure 3(b)) and a cell scratch assay (Figure 3(c)) were carried out. Exposure of MMECs to H/R decreased capillary-like-structure formation and EC migration; however, the exogenous anti-pro-BDNF antibody significantly enhanced H/R-induced migration of (and capillary-like-structure formation by) MMECs. Taken together, these data indicate that pro-BDNF was required for H/R effects in MMECs exposed to HG.

### 3.4. A Proapoptotic Protein Is Involved in the Regulation of Pro-BDNF Expression after H/R Injury in MMECs

**Figure 4:**
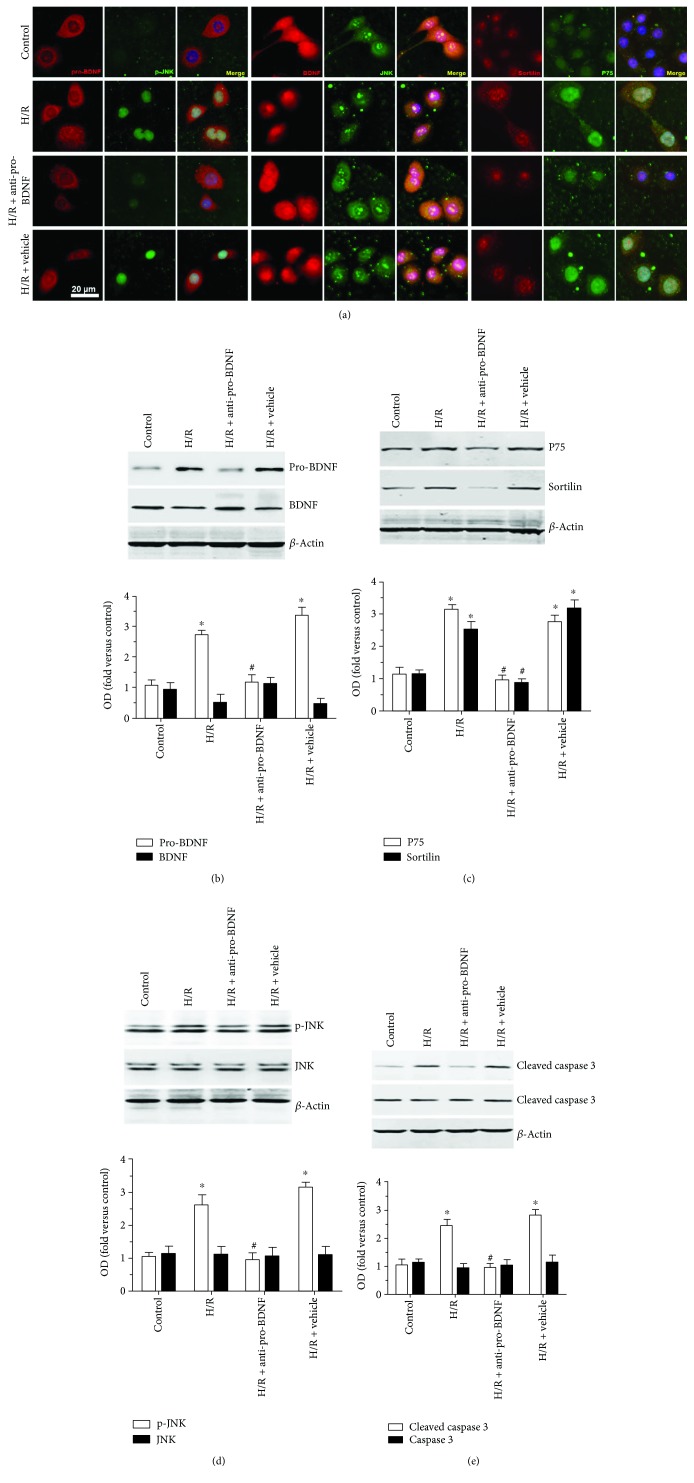
Effects of the anti-pro-BDNF antibody on the expression of p75^NTR^ and sortilin and apoptosis-related proteins. (a) Representative immunofluorescent images of pro-BDNF (red, first column), p-JNK (green, second column), BDNF (red, fourth column), JNK (green, fifth column), sortilin (red, seventh column), and p75^NTR^ (green, eighth column) in groups control, H/R, H/R + anti-pro-BDNF, and H/R + vehicle. (b–e) Representative Western blots and quantitative analysis of pro-BDNF, BDNF (b), p75^NTR^, sortilin (c), JNK, p-JNK (d), caspase 3 and cleaved-caspase 3 expression (e) in response to different treatments. All the data were normalized to *β*-actin, and fold changes are expressed in relation to the control group. Exposure of MMECs to H/R resulted in significantly higher expression levels of pro-BDNF, p75^NTR^, and sortilin and in activation of JNK and caspase 3 as compared with MMECs maintained under normal conditions (control). Nonetheless, there were no significant differences in BDNF, JNK, and caspase 3 expression levels after H/R. Treatment with the anti-pro-BDNF antibody significantly reversed the increase in the protein expression of pro-BDNF, p75^NTR^, sortilin, p-JNK, and cleaved caspase 3 in MMECs after exposure to HG and H/R (H/R + anti-pro-BDNF). The data were subjected to one-way ANOVA. The error bars represent SEM. ^∗^*P* < 0.05 as compared with the control group; ^#^*P* < 0.05 as compared with the H/R or H/R + vehicle group.

To elucidate the molecular mechanisms behind the action of pro-BDNF under HG and H/R conditions, experiments were performed on several markers of apoptosis by immunostaining (Figure 4(a)) and Western blotting (Figures 4(b)–4(e)). Colocalization of p75^NTR^ and sortilin in the cell membrane was observed in all groups. H/R led to increased p-JNK translocation to the nucleus. Exposure of MMECs to H/R caused significantly higher expression levels of pro-BDNF, p75^NTR^, sortilin, p-JNK, and cleaved caspase 3 as compared with MMECs maintained under normal conditions (*P* < 0.05). By contrast, there were no significant differences in BDNF, JNK, and caspase 3 expression levels after H/R. Of note, treatment with the anti-pro-BDNF antibody significantly reversed the increase in the protein expression of pro-BDNF, p75^NTR^, and sortilin and inhibited the activity of JNK and caspase 3 in MMECs after exposure to HG and H/R. Taken together, these data indicate that p75^NTR^ and sortilin and activation of JNK and caspase 3 are associated with the H/R-induced cellular injury.

## 4. Discussion

Hyperglycemia, a common feature of both type 1 and type 2 diabetes, is a key factor that contributes to the development of DM-related vascular disease and notably microvascular disease [39]. EC dysfunction and apoptosis have proved to play a vital role in the development of MIRI [40]. In the present study, no changes in cell migration and capillary-like-structure formation occurred, and no cell apoptosis was induced in MMECs cultured in DMEM high-glucose complete medium. However, in response to HG and H/R, MMECs showed increased levels of apoptosis and reduced migration and capillary-like-structure formation, suggesting that H/R resulted in MMEC injury. It is worth noting that pro-BDNF protein expression increased in the H/R-treated MMECs. Based on these results, we hypothesized that pro-BDNF might participate in the H/R-induced EC dysfunction and apoptosis.

One research group [41] reported that BDNF protects from cardiac dysfunction after myocardial infarction. Other researchers [17] found that BDNF protects human vascular ECs from apoptosis. In the present study, overexpression of pro-BDNF had proapoptotic effects on MMECs, but the neutralizing antibody to pro-BDNF significantly attenuated this apoptosis and the reduction in EC migration and capillary-like-structure formation by MMECs after exposure to HG and H/R. Consistent with the results obtained elsewhere [42–44], these data prove that pro-BDNF contributes to H/R-induced cell injury.

Pro-BDNF shows high-affinity binding to sortilin and performs its biological functions by acting on its receptors: p75^NTR^ and sortilin [45]. Some studies have indicated that the JNK pathway contributes to the growth-inhibitory effect and apoptosis of ECs and that inhibition of JNK activation protects cardiomyocytes from I/R injury [46–49]. Pro-NGF/p75NTR/sortilin signaling increases JNK signaling [50]. Furthermore, cleavage-resistant pro-BDNF mutant (CR-pro-BDNF) treatment resulted in a rapid phosphorylation of JNK which are involved in p75NTR-induced apoptosis and an earlier appearance of active caspase 3 in cerebellar granule neurons [51]. Pro-BDNF has also been proved to be a proapoptotic ligand for sympathetic neurons and could induce neuronal apoptosis via activation of a receptor complex of p75NTR and sortilin [24]. In the present study, the data on MMECs revealed that H/R, which enhanced pro-BDNF protein expression, induced P75^NTR^ and sortilin protein expression and increased activation of JNK and caspase 3. In contrast, the anti-pro-BDNF antibody significantly reversed these effects. Collectively, our data suggest that pro-BDNF exerts a proapoptotic effect against myocardial I/R injury at least in part through the regulation of p75^NTR^-sortilin signaling and activation of JNK and caspase 3.

Diabetic nephropathy is a serious microvascular complication of DM; H/R promoted oxidative stress in NRK-52E cells exposed to HG accompanied by increased levels of Nrf2 and HO-1 protein expression [32, 46]. High glucose has also been proved to increase the permeability of cardiac microvascular endothelial cells. Thus, the question of whether other molecular mechanisms contribute to the effect of pro-BDNF on H/R is an intriguing one and merits further investigation.

## 5. Conclusion

In summary, the major finding of our study is that inhibition of pro-BDNF may exhibit a beneficial effect against H/R by promoting MMEC migration and capillary-like-structure formation. Moreover, these effects are at least in part related to the decrease in MMEC apoptosis through p75^NTR^-sortilin-mediated activation of JNK and caspase 3. Our results may facilitate future studies on the therapeutic implications of pro-BDNF in the treatment of MIRI.

## Data Availability

The data used to support the findings of this study are available from the corresponding author upon request.

## Abbreviations

BDNF: Brain-derived neurotrophic factor
MMECs: Myocardial microvascular endothelial cells
H/R: Hypoxia/reoxygenation
DM: Diabetes mellitus
CMECs: Cardiac microvascular endothelial cells
NGF: Nerve growth factor
TNF-*α*: Tumor necrosis factor *α*
JNK: c-Jun N-terminal kinase
MIRI: Myocardial ischemia/reperfusion injury.
